# Characteristics, Barriers, and Facilitators of Virtual Decision-Making Capacity Assessments During the COVID-19 Pandemic: Online Survey

**DOI:** 10.2196/60574

**Published:** 2024-11-25

**Authors:** Lesley Charles, Eileen Tang, Peter George Jaminal Tian, Karenn Chan, Suzette Brémault-Phillips, Bonnie Dobbs, Camelia Vokey, Sharna Polard, Jasneet Parmar

**Affiliations:** 1 Division of Care of the Elderly Department of Family Medicine University of Alberta Edmonton, AB Canada; 2 Department of Occupational Medicine University of Alberta Edmonton, AB Canada; 3 Library Services Covenant Health Edmonton, AB Canada

**Keywords:** decision making capacity, mental competency, aged, mobile applications, mobile phone, Canada, covid-19, pandemics, dementia, survey, virtual capacity assessment, characteristics, barriers, facilitators, virtual decision making, assessment

## Abstract

**Background:**

With a growing older adult population, the number of persons with dementia is expected to rise. Consequently, the number of persons needing decision-making capacity assessments (DMCA) will increase. The COVID-19 pandemic has impacted how we deliver patient care including DMCAs with a much more rapid shift to virtual assessments. Virtual DMCAs offer patients and health care professionals distinct advantages over in-person delivery by improving reach, access, and timely provision of health care. However, questions have arisen as to whether DMCAs can be effectively conducted virtually.

**Objective:**

This study aimed to determine the characteristics, barriers, and facilitators of conducting virtual DMCA during the COVID-19 pandemic.

**Methods:**

We conducted an online survey among health care providers who perform DMCAs in Alberta from March 2022 to February 2023. The survey consisted of 25 questions on demographics, preferences, and experience in conducting DMCAs virtually, and risks and barriers to doing virtual DMCAs. The data were analyzed using descriptive statistics.

**Results:**

There were 31 respondents with a mean age of 51.1 (SD 12.7) years. The respondents consisted of physicians (45.2%, 14/31), occupational therapists (29%, 9/31), and social workers (16.1%, 5/31), with a majority (93.6%, 29/31) based in Edmonton. The mean number of years of experience conducting DMCAs was 12.3 (SD 10.7), with a median of 8 DMCAs (IQR 18.5) conducted per year. Most respondents conduct capacity interviews, with a majority (55.2%, 16/29) being associated primarily with acute care services. Furthermore, 54.8% (17/31) were interested in conducting DMCAs virtually; however, only 25.8% (8/31) had administered DMCAs virtually. Barriers and facilitators to virtual DMCAs relate to patients’ characteristics and environment (such as communication difficulties, hearing or visual impairment, language barriers, ease of use of technology, or cognitive impairment), technology and technical support (need for technical support in both the client’s and assessor’s sides, the unreliability of internet connection in rural settings, and the availability of high-fidelity equipment), and assessors’ ability to perform DMCA’s virtually (ability to observe body language, interact with the client physically when needed, and build rapport can all be affected when conducting a DMCA virtually). In terms of implications for clinical practice, it is recommended that the patient or caregiver be familiar with technology, have a stable internet connection, use a private room, not be recorded, use a standardized assessment template, and have a backup plan in case of technical difficulties.

**Conclusions:**

Conducting DMCAs virtually is a relatively infrequent undertaking. Barriers and facilitators to adequate assessment need to be addressed given that virtual assessments are time-saving and expand reach.

## Introduction

As populations around the globe are rapidly aging, a greater percentage of individuals require geriatric care [1]. Telemedicine offers a potential solution to enhancing access to health care services by delivering health care remotely. Telemedicine may benefit and be cost-efficient for those living in rural settings or experiencing mobility limitations [1-3]. Decision-making capacity assessments (DMCAs) are increasingly being conducted among aging individuals experiencing cognitive changes. Questions have arisen as to whether DMCAs can be effectively conducted virtually. Previous studies have signaled that teleneuropsychological assessments of older patients with cognitive disorders are equally effective as in-person assessments [4-6], with reports of high patient satisfaction and preference for virtual assessments over an in-person visit to a remote clinical center [7,8]. Physical distancing requirements necessitated by the COVID-19 pandemic set the context specifically for virtual delivery of DMCAs to be explored. DMCAs can cover the following domain or domains (ie, health care, accommodation, choice of associates, social activities, legal affairs, employment, education, vocational training, and finances). The DMCAs referred to in this paper can cover any of the aforementioned domains.

Although virtual DMCAs are relatively new, a few studies outline the process. The process includes videoconference technology delivered over a tablet, smartphone, or computer. Other studies use an innovative web-based system that stores notes, client records, and photographs securely. The health care professional conducting a virtual DMCA can be supported by a caseworker who is physically present with the patient. The caseworker can be instructed by the evaluating geriatrician to help facilitate the assessment [9]. The caseworker’s responsibilities include but are not limited to, bringing necessary equipment for the videoconference, establishing an audiovisual connection, administrating relevant cognitive assessment instruments, uploading completed information into the web portal, and facilitating viewing of living conditions [10].

Virtual delivery of DMCAs has also been reported by Factora and Hashmi [11] based on a capacity evaluation program. Geriatricians are connected to patients through a videoconference platform. A nurse, present with the patient, supports the assessment, including obtaining vital signs, conducting a medication review with pill count, facilitating cognitive evaluation, and resolving technological barriers faced by the patient [11]. These programs demonstrate the viability and applicability of virtual DMCAs.

While being a novel and potentially transformative delivery method, virtual assessment does not come without obstacles. Disadvantages of virtual neuropsychological assessments include videoconferencing, and relying on Wi-Fi or cellular connection. Issues with connectivity or audiovisual quality can hinder the quality of virtual DMCAs for both the clinicians and the person being assessed [2,9,12]. A patient’s sensory-motor and visual challenges can also disrupt their ability to pick up nonverbal cues, making virtual administration of a DMCA and determination of a patient’s actual capacity difficult [7,9,12]. Solutions for these matters are fortunately available. Caseworkers (often social workers) or nurses physically with the patient can bring and set up devices and connections to ensure the quality of the videoconference [10]. Sensory-motor impairments can be accommodated using multiple adjustments including the provision of glasses, hearing aids, interpreters, and amplifiers, and written verbal instructions so that patients can best participate in the assessment [9]. According to Halphen et al [9], connection and sensory limitations are usually not significant enough to warrant switching the DMCAs from virtual to in-person, with less than 1% of DMCAs being aborted.

Virtual DMCAs offer patients and health care professionals distinct advantages over in-person delivery. Virtual DMCAs improve reach, access, and timely provision of health care as they eliminate geographic barriers that could hinder physician availability [1,4]. Patients across large geographic areas can access a central hub of strongly resourced and competently trained physicians and other health care providers through videoconference and enhance the uniformity of high-quality health care delivery [2]. Virtual DMCAs also enable health care providers to reallocate travel time to more clinical tasks such as addressing polypharmacy and increasing communication with tertiary medical care [8,12]. In addition, virtual DMCAs can aid patients in enhancing their quality of life. A recent study by Factora and Hashmi [11] examining the impact of virtual DMCAs on the provision of community support and living outcomes found that 89% of individuals were successfully provided with community services. It also found that the number of individuals who could access routine primary care increased from 43% to 81% as more people were newly connected, and 52% of people were appointed guardianship as a direct result of receiving a virtual DMCA. Most importantly, being connected to these services helped individuals remain living at home. There is a common perception that engagement in a virtual DMCA increases the chance of patient being assigned guardianship and placed in a long-term care facility. This misconception, however, is not the case [11]. Overall, virtual DMCAs have been shown to provide good outcomes for both health care professionals and patients and align with their best interests.

The COVID-19 pandemic impacted how patient care, including DMCAs, was delivered with a much more rapid shift to virtual assessments. The objective of this study was to determine the characteristics, barriers, and facilitators of conducting virtual DMCAs during the COVID-19 pandemic. The DMCAs will have considered any of the domains of health care, accommodation, choice of associates, social activities, legal affairs, employment, education, vocational training, and finances.

## Methods

### Survey Design and Participants

This was an exploratory online survey using the SurveyMonkey Platform. We used a purposive sampling of health care professionals who conducted decision-making capacity assessments in Alberta; all professionals who currently or previously provided DMCAs were eligible. We used the contacts and networks of 4 authors (LC, SB-P, JP, and KC) to send email invitations to participate in the survey voluntarily and without incentives. The survey invitation was disseminated at 4 time points (March 2022, December 2022, January 2023, and February 2023) through the Alberta College of Family Physicians, Alberta Medical Association, the University of Alberta’s Department of Family Medicine and Divisions of Care of the Elderly and Geriatric Medicine, Edmonton Zone Medical Staff Association, Edmonton Zone Decision-making Capacity Committee, Office Public Guardian and Trustee and partners associated with the authors (LC, SB-P, JP, and KC are family physicians who have been conducting DMCA workshops across Alberta for more than 15 years; SB-P is an occupational therapist who has also conducted DMCA workshops).

The invitations were emailed to specific eligible individual contacts. The invitations were also emailed to groups that had potential respondents; as such, the total number of invitees could not be ascertained, and the response rate could not be determined. The invitees represent professionals from various settings: community, home living, supportive living, long-term care, acute care, and rehabilitation.

The survey questionnaire consisted of 25 consecutive questions, in a fixed sequence; the questions were a combination of multiple-choice, yes-no, ranking, Likert-like rating, and open-ended questions. The first of 3 parts of the questionnaire asked about age, occupation, training and experience in DMCA, and occupational settings. The second part asked about preferences and experience in virtual DMCAs. The third part asked about the risks, barriers, and facilitators to doing virtual DMCAs. Refer to Multimedia Appendix 1 for the questionnaire. Completion of this anonymous survey was deemed as implied consent.

### Ethical Considerations

The study was approved by the University of Alberta Health Research Ethics Board (study ID Pro00110017). The participants were provided with an online information letter and consent statements. Consent was implied when the participants proceeded with and submitted the survey. The survey was anonymous with no identifiers were collected. No compensation was provided for participation.

### Data and Analysis

The survey collected data on 25 variables. Descriptive statistics were used to describe the variables quantitatively using Microsoft Excel 2019. Responses to open-ended questions were grouped into categories.

## Results

### Overview

There were 31 respondents with a mean age of 51.1 (SD 17.7) years. The respondents consisted of physicians (45.2%, 14/31), occupational therapists (29%, 9/31), and social workers (16.1%, 5/31), with most respondents working in the Edmonton area (29/31, 93.6%). The mean number of years of experience conducting DMCAs was 12.3 (SD 10.7), with a median of 8 DMCAs (IQR 18.5) conducted per year. Most respondents conducted capacity interviews, with a majority (55.2%, 16/29) being associated primarily with acute care services (Table 1).

**Table 1:** Characteristics of survey respondents (n=31; DMCA^a^ providers in Alberta, Canada; 2022-2023).

Characteristics			Values
**Age (years)**			
	Mean (SD)	51.1 (12.7)	
	Median (IQR)	50 (15)	
**Occupation, (n=31), n (%)**			
	Physician	14 (45.2)	
	Occupational therapist	9 (29)	
	Social worker	5 (16.1)	
	Other	3 (9.7)	
**Previous training in DMCA, (n=31), n (%)**			
	Workshops	13 (41.9)	
	Talks	3 (9.7)	
	None	2 (6.5)	
	Refreshers	1 (3.2)	
	Other^b^	12 (38.7)	
**Years Conducting DMCAs**			
	Mean (SD)	12.3 (10.7)	
	Median (IQR)	10 (8)	
**Number of DMCAs conducted per year**			
	Mean (SD)	14.6 (15)	
	Median (IQR)	8.5 (18.5)	
Conducts capacity interviews, (n=30), n (%)			26 (86.7)
**Work settings ranked first in terms of amount of work done, (n=29), n (%)**			
	Acute care	16 (55.2)	
	Outpatient	4 (13.8)	
	Home living	3 (10.3)	
	Supportive living	2 (6.9)	
	Rehabilitation	2 (6.9)	
	Family practice	2 (6.9)	

^a^DMCA: decision-making capacity assessment.

^b^Other training specified: residency or specialty training, observation, practice, certification course, booster sessions, readings, and literature review.

Of the respondents (n=31), 54.8% (17/31) were interested in conducting DMCAs virtually and 25.8% (8/31) reported having conducted DMCA virtually. A respondent specified the use of telehealth with in-person support from licensed Practical Nurses, Registered Nurses, and Nurse Practitioners. A capacity interview worksheet and capacity assessment worksheet were not used. The same respondent reported being “Very Comfortable” doing virtual DMCA, however, noted that the virtual nature made the assessment difficult.

In contrast, 45.2% (14/31) of survey respondents declined to conduct DMCAs virtually, commenting that virtual DMCAs were inadequate and missed nuances, in-person DMCAs were better at establishing rapport, face-to-face interactions were preferred, challenges with technology and sensory impairments were worrying, and access to private spaces to conduct virtual assessments was a concern.

Reasons underlying hesitancy to conduct virtual DMCAs were explored by asking respondents to identify potential risks and barriers they associated with virtual DMCAs. Barriers and facilitators to virtual DMCAs were grouped according to 3 broad categories: the client’s side, technology, and assessor’s side. Refer to Table 2 for the text responses. We combined the answers to risks and barriers together and labeled these as barriers. We combined the answers to mitigants and facilitators together and labeled these as facilitators. The rationale for combining was that the answers were similar.

**Table 2:** Barriers and facilitators to conducting virtual decision-making capacity assessments (DMCAs) as perceived by DMCA providers, Online Survey, Alberta, 2022-2023.

	Barriers	Facilitators
Client’s side	Client’s communication difficulties may be amplified (or helped) with technology. Client’s hearing impairment, visual impairment, and understanding may affect communication. Clients may have language barriers. Client may be unable to perform or interact at their best. Client may be uncomfortable, or experience added stress from the technology. Client will receive restricted sensory input. Client may be disengaged or have difficulty with attention. Clients with cognitive impairment may experience confusion. Clients with mental health issues may experience increased agitation. Other people may influence or interfere with the assessment (eg, coaching, coercion, distraction). Client’s privacy could not be ensured. Client’s environment could not be controlled. Clients may have issues with the ability to use or afford technology. Client may have issues with the acceptability of virtual methods.	Having an assistant on site to be the eyes and ears, to provide more collateral information, to repeat questions, to mitigate concerns, and to assist throughout the assessment. They have to be nonprejudicial, trained, and experienced. [Note: This may create a privacy concern.] Ensuring that the patient is comfortable with the virtual assessment. Ensuring that no person other than support is with the patient. Ensuring no coaching. Minimizing distractions and ensuring quiet in the assessment room. Ensuring clients’ hearing and visual needs are met. Having more than one virtual visit to complete the interview. Allowing time for the client to familiarize with the technology. Precall confirmation may help. Having met the client or family before or knowing the client or family in advance. Consent to everyone in the room. Prohibition of recording of session. Caregiver, family, or friends being present. But must limit bias of family or friends during the interview. Ensuring equity of utilities such as telephone and internet. Engagement of people of all ages with accessible technology to reduce fear. Ensuring that there is no acute illness ongoing. Location should be well-lit, quiet, private, and relaxing or comfortable.
Technology	Use of technology may require support (eg, setting up and troubleshooting) on both client’s and assessor’s side. Unreliability of internet in rural settings. Technology may foster miscommunication. Security of internet connection. Adequacy of setup and high-fidelity equipment. Lack of availability of technology in remote areas. Discomfort with or lack of preference for technology. Confidentiality and privacy.	Having a trustworthy person helping to operate, optimize, and support the environment or technology (Note: This may create a privacy concern.) Ensuring good audiovisual equipment; with high-quality resolution video chatting. Having a secure videoconferencing platform, with no time limitations Avoiding virtual backgrounds. Using a platform with a camera and microphone. Enhanced security features are offered by electronic medical records. Smartphone enhancements.
Assessor’s side	Assessor may not get a true sense of the client. Assessor cannot observe body language (nonverbal communication) to aid the client navigate the assessment. Assessor may miss physical cues. Assessor could not interact with the client physically, if necessary. Assessor has less ability to develop a relationship or trust, identify mood or anxiety, and get the client to do practical tasks. Assessor has reduced rapport building. Assessor may have more difficulty in managing distractions or redirecting the client. Assessor could not ensure the psychological safety of the client. Assessor could misinterpret a slight time lag in videoconferencing as slow processing speed. Assessor has limited exposure to patient function and caregiver dynamics. Assessor may have difficulty overcoming a client’s communication disorders. Inability to use physical tools on clients. Decreased control over the client’s environment Questions on the acceptability of virtual methods. Time-consuming. Too mechanical. Lack of training and familiarity with virtual assessment. Comfort with technology. Difficulty assessing patient function with caregiver. Difficulty setting up appointments.	Having an emergency plan in place before starting. Having a supporting video clip of unstructured time. Education or training modules. Previous interactions with caregivers or agents. Developing templates for important factors, aspects, and observations. Availability of medical or collateral information to assist with the assessment. A small team with a lot of experience doing virtual DMCAs. Development of a checklist to ensure barriers are addressed in advance planning.

### Barriers and Facilitators From the Client’s Side

Respondents commented that barriers to virtual DMCA could arise from the client’s characteristics, such as communication difficulties, hearing or visual impairment, language barriers, ease of use of technology, or cognitive impairment. Barriers could also arise from the client’s environment such as distraction, lack of privacy, and potential for coercion or coaching. Several facilitators were suggested to address these barriers, such as having an assistant to address the client’s needs and help in the assessment, ensuring privacy, and having more than one virtual visit.

### Barriers and Facilitators in Technology

Barriers included the need for technical support on both the client’s and assessor’s sides, the unreliability of internet connection in rural settings, and the availability of high-fidelity equipment. To address these barriers, the following were suggested: provision of technical support, good equipment, and ensuring privacy.

### Barriers and Facilitators From the Assessor’s Side

The most notable barriers identified related to difficulty assessing a client virtually. The assessor’s ability to observe body language, interact with the client physically when needed, and build rapport can all be affected when conducting a DMCA virtually. Suggestions to address these barriers included developing checklists, providing training, and the availability of collateral information to assist with the assessment.

## Discussion

### Principal Findings

This study explored health care professional’s perceptions on the virtual delivery of DMCAs. Overall, 45.2% (14/31) of survey respondents were not interested in conducting DMCAs virtually. This could be due to perceptions that in-person delivery better enhances rapport, ease of access to nuances, accommodation for technology and sensory challenges, and management of privacy issues. Other studies in the United States have reported that virtual DMCAs are as feasible and effective as in-person DMCAs and that medical staff can benefit from performing virtual DMCAs over in-person ones as they eliminate geographic barriers to health care and improve time savings for more clinical tasks as discussed further in the introduction [1,2,4-6,8,12].

Potential risks and barriers associated with virtual DMCAs were identified. The respondents highlighted several risks associated with virtual DMCAs, including potential technological glitches. Concern regarding audiovisual quality was also found in other studies [9]. In addition, respondents identified various barriers that could hinder the successful implementation of virtual DMCAs including issues with cost, technology, and connectivity. The potential risks and barriers that respondents brought up in the survey also corroborated with other studies that conclude similar potential issues, suggesting that people are generally aware of common hazards when conducting virtual DMCAs [1,2,6,7,9,10,12].

After establishing risks and barriers, respondents did offer some insightful suggestions, methods of mitigation, and potential facilitators to counteract issues associated with virtual DMCAs. To mitigate respondents recommended: having a trustworthy support system to operate technology and ensure access to a private quiet room for assessment, prioritizing a stable internet connection, using proper encryption to secure the videoconference, refraining from using virtual backgrounds, refraining from recording sessions to uphold confidentiality and establishing a backup plan in case of technical difficulties, creating standardized assessment templates to ensure consistency, and maintaining a high video and audio quality. Specifically, facilitators with technical expertise, people who could set up and maintain technology during virtual DMCAs, were emphasized as many respondents believed that having access to experienced technical support is crucial for the success of virtual DMCAs. Similarly, these responses are supported by literature as other studies have implemented these suggestions and facilitators in their versions of virtual DMCAs, such as having a caseworker and/or nurse in-person to operate technology and perform some of the physical aspects of the assessment which the clinician is unable to virtually [9-11]. While respondents offered a range of insightful suggestions to address the challenges associated with virtual DMCA assessments, there was an underlying impression that some individuals expressed uncertainty regarding these suggestions, evident through the use of question marks in their responses or by phrasing their suggestions in a hypothetical and hesitant manner. This cautious attitude amongst respondents suggests that while the provided suggestions hold potential solutions, there remains a degree of ambiguity or skepticism among certain respondents regarding the effectiveness of virtual DMCAs. Recommendations for clinical practice. It is recommended that the patient or caregiver be familiar with technology, have a stable internet connection, use a private room, not be recorded, use a standardized assessment template, and have a backup plan in case of technical difficulties.

This study adds to the literature as it specifically looks at the rapid transition to virtual DMCA during the COVID-19 pandemic. It also expands on barriers and facilitators to conducting virtual DMCAs that is in the limited existent literature. Other studies comment on barriers and facilitators but that has not been the main focus of their study.

### Limitations

This study has several limitations. First, the respondents were from a single province and the results may not be applicable in other provinces or countries. Legislation related to capacity varies by province. However, the key concepts of conducting a DMCA are transferable and thus should not have affected the results. Second, virtual DMCAs are not commonly carried out. This limited the responses to perceptions rather than responses based on lived experience. Third, the responses to open-ended questions were scarce, ranging to a word up to 5 sentences. A more detailed qualitative study using focus groups would complement this survey.

### Conclusions

Conducting DMCAs virtually is a relatively infrequent undertaking. Barriers and facilitators to virtual DMCAs relate to patients’ characteristics and environment, technology and technical support, and assessors’ ability to perform DMCA’s virtually. Barriers and facilitators to adequate assessment need to be addressed given that virtual assessments are time-saving and expand reach.

## Appendix

Multimedia Appendix 1 The questionnaire.

## Abbreviations

DMCA: decision-making capacity assessment

## Data Availability

The data analyzed during this study are available from the corresponding author on reasonable request.
