# Supplementary material for: Characteristics, Barriers, and Facilitators of Virtual Decision-Making Capacity Assessments During the COVID-19 Pandemic: Online Survey
Source: JMIR Form Res. 2024 Nov 25;8:e60574. doi: 10.2196/60574 (PMC11629031; doi:10.2196/60574)
Supplement: Multimedia Appendix 1 [file formative_v8i1e60574_app1.docx]

### Appendix A. The Questionnaire

1. **What is your age?**

[Open-ended question]

1. **What is your occupation (e.g., nurses, social worker, physician)?**

[Open-ended question]

1. **What is your prior training in DMCA? Please provide details?**

- None
- Talks
- Refreshers
- Workshops
- Other, please specify:

1. **What is your prior experience in conducting DMCAs, please detail:**

[Open-ended question]

1. **How many years have you been conducting DMCAs?**

[Open-ended question]

**On average, how many DMCAs do you perform a year?**

[Open-ended question]

1. **Do you conduct capacity interviews?**

- Yes
- No

1. **In which AHS Zone do you work in?**

- Calgary Zone
- Central Zone
- Edmonton Zone
- North Zone
- South Zone
- Other, please specify:

1. **Among the work setting below, rank the settings that you work in terms of the amount of work done (with 1 being the most amount of work done):**

Home Living Rank: ___

Supportive Living Rank: ___

Facility Living Rank: ___

Acute Care Rank: ___

Outpatients Rank: ___

Family Practice Rank: ___

Rehabilitation Rank: ___

1. **Are you interested in conducting DMCA virtually?**

- Yes
- No

1. **If you have declined to conduct DMCA virtually, what were the reasons for the refusal.**

- Not applicable

1. **Have you conducted a DMCA virtually?**

- Yes [logic: go to question 12]
- No [logic: go to question 21]

[Logic: If the answer to question 11 is yes]

1. **Prior to the COVID pandemic, how many virtual DMCAs have you performed?**

[open-ended]

1. **During the COVID pandemic (March 2020 – present), how many DMCAs have you performed?**

[open-ended]

1. **Which tool or technology did you use in performing DMCAs virtually?**

- Telephone
- Zoom
- Other, please specify:

1. **Which disciplines did you involve in the virtual DMCAs?**

- Geriatrics
- Medicine (Physicians)
- Nursing (Licensed Practical Nurses, Registered Nurses, Nurse Practitioners)
- Occupational Therapy
- Physical Therapy
- Psychiatry
- Psychology (Psychologist)
- Social Work
- Other, please specify:

1. **Did you use of the Capacity Assessment Worksheet?**

- Yes
- No

1. **Did you use of the Capacity Interview worksheet? Yes/No**

- Yes
- No

1. **Rate your comfort with assessing DMCA virtually**

| **Very Uncomfortable** | **Uncomfortable** | ****  **Neutral** | ****  **Comfortable** | ****  **Very Comfortable** |
| --- | --- | --- | --- | --- |

1. **Rate the impact of performing DMCAs virtually on how DMCA is usually done.**

| ****  **Made DMCAs**  **Very Difficult** | ****  **Made DMCAs**  **Difficult** | ****  **Neutral** | ****  **Made DMCAs**  **Easier** | ****  **Made DMCAs**  **Much Easier** |
| --- | --- | --- | --- | --- |

**Please describe the impact of the virtual method on how DMCA is done.**

[open-ended]

1. **When in-person care would be more available, rate your likelihood of continuing to do DMCAs virtually.**

| ****  **Very Unlikely** | ****  **Unlikely** | ****  **Neutral** | ****  **Likely** | ****  **Very Likely** |
| --- | --- | --- | --- | --- |

**Please describe your preference (open field):**

[open-ended]

[Logic: Follows question #20; or Follows question #11 if the answer is yes]

1. **What are the risks of performing DMCA virtually versus in person?**

[open-ended]

1. **How could the risks of performing DMCAs virtually be mitigated?**

[open field]

1. **What are barriers to conducting DMCA virtually?**

[open-ended]

1. **What are facilitators to conducting DMCA virtually (open field)?**

[open-ended]

1. **Please write suggestions for performing virtual DMCAs successfully.**

[open-ended]

Thank you for your participation.
